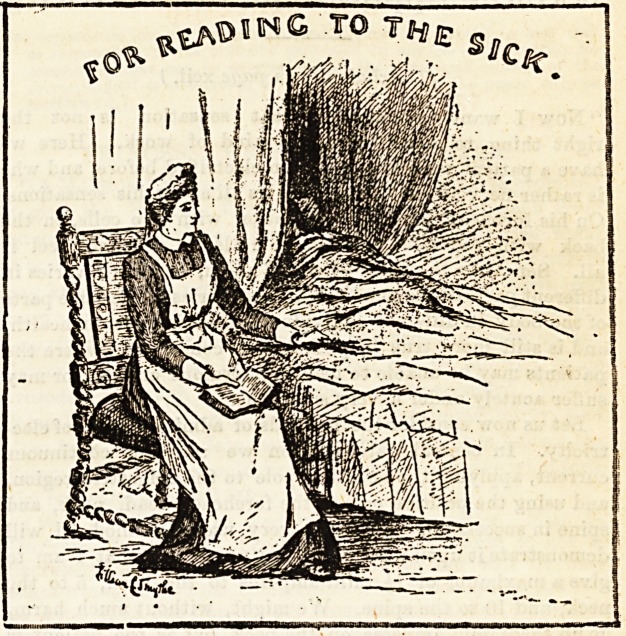# Extra Supplement—The Nursing Mirror

**Published:** 1891-01-31

**Authors:** 


BEING THE EXTRA NURSING SUPPLEMENT OF "THE HOSPITAL" NEWSPAPER.

Contributions for this Supplement should be addressed to the Editor, THE HOSPITAL, 140, Strand, London, W.C., and should have the word "Nursing" plainly written in left-hand top corner of the envelope.

### En Passant.

RURAL NURSING ASSOCIATION.—Lord Fitzwilliam presided lately at an important meeting at York for the promotion of the branch of the above association in that county. Mrs. J. C. Dundas read an excellent paper on "Nursing in Rural Districts," pointing out the necessity for nurses to have a fair amount of training, and a fair amount of intelligence, lest their efforts should be followed by disasters.

PERTH NURSING SOCIETY.—The sixth annual meeting was held on January 13th, Lord Provost Wilson presiding. The society is doing satisfactorily, and has decided not to affiliate with the Jubilee Institute. There is at present a superintendent and an assistant nurse, and Mr. Cowan called on one of the young ladies of Perth to come forward and offer herself for training for the society. Over 260 cases were attended last year. The debt on the Home has been paid off, and the rich of the town continue to be interested in the work, and the poor highly appreciate its benefits.

A MIDWIFE IN TROUBLE.—At an enquiry concerning the death of Mrs. Spicer, held last week, Mrs. Elizabeth Sarah Berry, who acted as a midwife, was committed on trial for manslaughter. Mrs. Berry had attended a patient suffering from puerperal fever; the doctor cautioned her not to attend another confinement for a month, in spite of which warning she attended Mrs. Spicer, who subsequently died of puerperal fever. Mrs. Berry said she was not certificated; she did not know what puerperal fever was; she had attended about nine hundred midwifery cases. Such awful ignorance and recklessness caused quite a sensation in court. And yet there are people who have the audacity to oppose the Midwives Registration Bill. We are glad the jury brought in the verdict they did, though we are sorry for Mrs. Berry, whose sin was evidently one of ignorance and denseness.

A GENTLEMANLY DISCUSSION.—The fortnightly meeting of the Sanitary Committee of the Oldham Corporation lately had the following point before them: The Matron of the Westhulme Hospi[illegible] suffered on the Wednesday night from hyper-pyrexia, her [illegible]mperature going up to 108 deg., and remaining at that height for a number of hours. The medical officer of health called in Dr. Dreschfeld in consultation; the debated point was, Who was to pay the fee of four guineas for the consultation? That the medical officer did right in such an extreme case to call in another medical man no one with any knowledge of medicine can deny, nor was it to be wondered at that the Corporation should raise the question of payment of the extra fee. But there is a right way and a wrong way of doing things, and to estimate the feeling which animated the Board it is necessary to quote a few sentences from the debate: "Alderman Jackson warmly remarked that there seemed to be too much made of the nurses at the hospital nowadays—they were almost idolised from certain quarters, and they were getting more aristocratic than the nobility—one scarcely dared to approach a nurse now. It was nothing less than toadyism." Another remark made, was too blasphemous to be repeated here. The one good point to be seen in this disgraceful debate is the dignified attitude of Dr. Niven; and perhaps we ought to remark on the peaceable desires of the Chairman, who might advantageously have used more power.

KEEPING CHRISTMAS.—We must finally conclude our accounts of Christmas festivities with a few notes here. The medical staff, nurses, and friends of the Derbyshire Infirmary held a delightful musical evening last week. At Torbay Hospital an entertainment was given in No. 8 Ward, the first part songs and recitations, and the second part, a pleasantly-acted farce, by certain gentlemen who are too modest to desire their names to appear in print. At the Royal Hants County Hospital the usual dramatic performance was forsaken in favour of a concert, because of the unusual number of accident cases in the ward. There was the usual tree and pretty decorations of evergreens, flags, and fairy lights. A stand of elaborately-dressed dolls in fancy costume was much admired. As usual there was a large attendance of visitors in response to the invitations issued by the Lady Superintendent. All spent a most enjoyable afternoon.

SHORT ITEMS.—The Devonshire branch of the Rural Nursing Association has voted a grant to Chagford, to enable that village to start a district nurse.—We hope that the authorities of the Birkenhead Fever Hospital will have that building put in thorough repair; it is no use to do things by halves, and even trained nurses cannot work well in bad wards.—There are some other towns, especially one in the North of England, that would do well to inspect their infectious hospitals if they wish to avoid scandals.—The ladies of the Nelson Settlement, Blackfriars, propose to institute a parochial nurseship in memory of Miss Benson.—Seventeen young widows under the charge of Pundila Ramabai are being trained at Poona as hospital nurses.—The Queen of Roumania has given the profits arising from her literary labours to the Pen-Bron Children's Hospital.—We understand that the charge night nurse lately appointed at Manchester Eye Infirmary, formerly acted as laundress in that institution, and is untrained.

RISING TO THE OCCASION.—An epidemic of measles broke out in Ipswich in December, and what with sickness, and cold, and poverty, the poor children were suffering terribly. Miss Pye, of the Ipswich Nurses' Home, organised a fund for the relief af these cases, and while the epidemic was at its worst a tour of investigation was made three times a day by a competent nurse, and coal, milk, and cornflour were sent immediately to the cottages. This was followed by a systematic supply of soup—eggs and jelly in diphtheritic cases—milk, and rice puddings as needed. Each cottage had two bushels of coal and two bushels of coke weekly, for three weeks, and longer if needful. One hundred and thirty-nine families, with 419 sick children, had been helped. During the past fortnight, where the fathers were out of work owing to the frost, 200 loaves of bread had been given from the fund. The Rev. W. Berry writes to Miss Pye: "The people who have subscribed to your fund have done well in entrusting you with the distribution of their bounty. You have not only acted on my recommendation, but you have personally investigated some of the most serious cases, such as diphtheritic throats; and have thus been able to give advice and render help which was most useful. I think it is most important for a trained nurse to visit the homes of the poor in sickness, and direct them as to sanitary matters, &c. I do not ever remember being connected with any relief fund which was more judiciously administered or more needed at the time, and I must congratulate you on the happy thought which came to your mind to organise and carry out the undertaking, which was done, moreover, not only efficiently, but without any undue fuss."

### Lectures on Surgical Ward Work and Nursing.

By Alexander Miles, M.B. (Edin.), C.M., F.R.C.S.E.

Lecture XI.—THE SPRAY DRESSING *(continued).*

So much, then, for the spray, its principles, and its dangers. In what else does a spray dressing differ from another? Practically in nothing. You must be more careful to avoid draughts when the spray is being used, as any current of air will blow the vapour off the wound, and the chances of organisms reaching it are much increased. Therefore, all doors, windows, and ventilators should be closed, and *kept closed*, while the spray is going, no one entering or leaving the room if it can possibly be avoided. This renders it necessary that nothing be omitted from the dressing tray which can possibly be wanted. Never forget to open the ventilators again after the dressing is over.

On account of the vapour condensing, it is necessary to protect the patient and the bed by more macintoshes and dipped towels than in an ordinary dressing, and as many patients, especially children, object to the carbolic being blown in their faces, it is well to cover the head with a dry towel. There is still another set of precautions to be observed at a spray dressing: (1) Never open out a wound before the spray is going; a safe rule is "turn on the spray so soon as ever the bandage is cut." (2) Never allow anything to get between the spray and the wound, otherwise you interrupt the current, and permit of uncarbolised air gaining access. (3) Never shut off the spray till the wound is covered up; the rule should be, "so soon as the bandage is begun the spray to be stopped." (4) If by any chance the spray should give signs of failing during a dressing, when only one nozzle is in use, you must on no account turn on the other nozzle, in the hope that thereby you will improve matters. Doubtless you will increase the volume of steam, but it will only last half as long, so that while with one nozzle you might be able to finish your dressing with a weak though efficient spray; with two you will run short of steam before you are finished, and might as well be without altogether.

The Spray Stand (Down Brothers).

The Spray Stand.—This is a piece of apparatus which can easily be extemporised, but in wards where the spray is constantly used some form of properly made stand is employed. The main requisites are that it be steady, and that it be capable of being raised and lowered at pleasure to suit the height of the bed. This latter end is usually attained by means of a telescopic arrangement. Never attempt to alter the level of the stand without first removing the spray from it.

Let me summarise the precautions necessary for working the spray efficiently and safely: (1) Always fill the lamp, to begin with; (2) always fill the boiler two-thirds full with *hot* water; (3) always keep the carbolic jar filled; (4) always see that the steam is carbolised; (5) always provide against draughts in the ward; (6) never fill or even open the lamp while lighted; (7) never leave the lamp burning after dressing; (8) never allow the water in boiler to "boil out"; (9) never take out plug of boiler before water cools; (10) never turn spray directly on to patient; (11) never expose the wound without spray; (12) never allow anything to get between spray and wound; (13) never stop spray before bandage is begun; (14) never turn on second nozzle if spray fails; (15) never change the level of the stand with the spray on it.

Doubtless you will be inclined to say, after reading all these precautions, dangers, and sources of failure associated with the spray, "Is the benefit derived from the use of it worth all the bother and risk?" This question has been often asked, and variously answered. When Lister first introduced the spray its value was fully recognised, and it became almost universally used. At the same time, all other antiseptic precautions were increased, and the results became so good that many surgeons felt that, with efficient irrigation, the spray could be dispensed with, and for a time the pendulum of surgical opinion swung in the opposite direction, many abandoning its use. Some, however, remained faithful to it, and I question very much if their results do not justify them; but, of course, in the absence of statistical comparison, such an opinion cannot be insisted upon too far. Recently, however, in spite of its illustrious introducer publicly disowning it, the spray has again been more generally used. I cannot better state the case for the spray than by quoting an eminent abdominal surgeon, Mr. Knowsley Thornton, who says: "First, I believe it is useful during an operation in keeping a moist antiseptic atmosphere over everything, so that minute dry particles are immediately caught and moistened by a strong solution of a powerful germicide, while the hands of the operator and his instruments, as well as the tissues of the patient, are never allowed to become caked with dry blood or wound secretion, mixed with, and holding firmly, small dry particles from the atmosphere of the room; or, perhaps, the still more dangerous moist particles from the breath of those engaged in the operation or present as spectators. . . . This brings me to the second advantage derived from the steam saturated with carbolic" (as against plain steam). "It is urged that it is useless, because stronger solutions fail as germicides with certain potent germs, even when they are long exposed to their action. Those who argue thus forget one important element in surgery, *i.e.*, the vitality and resisting power of the tissues. Now I firmly believe that this vital action may be, and is, much aided when the germs of infection are delivered over to the tissues, weakened by being soaked in a strong antiseptic; perhaps this does not kill them, but it renders them a much easier prey to the active leucocytes."

*(To be continued.)*

### Nursing Medals and Certificates.

KENT NURSING INSTITUTION.

One of the oldest provincial nursing institutions is that at West Malling, which works under the above title. There is also a branch at work at Tunbridge Wells. It will be remembered by many of our readers that last autumn Princess Louise opened a bazaar in Knole Park on behalf of the Kent Nursing Institution, and that at the same time she presented the silver medal of the society to one of the nurses. All the nurses of this institution must be communicants of the Church of England, and they must be equally ready to nurse the rich or the poor. If they serve the institution faithfully for four years they receive the above medal in bronze; if they serve for eight years they are given a silver medal. There are only seven nurses who have received the silver medal, and their names are as follows: Nurses Holloway, Potter, Richardson, Carnell, Hall, Irwin, and Pennington.

### Sewing Competitions.

We have this week sent to Nurse Ethel Strachan, Sussex County Hospital, a cheque for two guineas, and to Nurse Ellen Tarrant, 33, Portman Square, a copy of Tennyson's works; being the prizes won in the invalid's jacket competition. So welcome have these jackets been to the institutions which received them, that we have determined to offer many more prizes for next Christmas, and we notify them now, so that our readers may be working for us all the year: (1) For the best pair of socks knitted by a nurse; (2) for the best pair of socks knitted by any Hospital reader; (3) for the best made flannel shirt; (4) for the best made flannel petticoat; (5) for the best made warm woman's bodice; (6) for the best made and best shaped dressing gown cut out and made by a nurse. Further particulars will be given later on.

### Appointments.

[It is requested that successful candidates will send a copy of their applications and testimonials, with date of election, to The Editor, The Lodge, Porchester Square, W.]

Holloway Infirmary.—In September, 1889, we chronicled the appointment of Miss Gertrude A. Wyld as Matron of the Chichester Infirmary, and ever since we have noted with pleasure how her sway there has resulted in better nursing. But Miss Wyld is ambitious, and now she has gone a step further and secured the post of Matron to the Infirmary, Archway Road, Upper Holloway, where there are 650 beds. We cannot do better than wish Miss Wyld equal success in her new sphere of work.

THE SUN.

Sunshine and light make much of the happiness of mankind, and there are few of us who do not love them dearly. We have had a long trial this winter of fog and gloom, and when the sun appeared for a few days there was great rejoicing. Unfortunately, in many hearts there is a like constant gloom and depression, which they do not ask, or even hope, that the Sun of Righteousness will remove. Others, again, light a flaring jet which they call pleasure, and expect by rioting and thoughtlessness to make their lives bright and jolly. Such mirth and laughter is but the crackling of thorns under a pot. They are soon burnt out, and the blackness is more dreary than ever.

We know that there is a remedy for this, but how to come at it is the question. We have become so bruised and battered with groping in the darkness, running sometimes against one obstacle and sometimes against another, that, sick and disheartened, our life seems almost squeezed out of us.

Take courage, the sun will surely appear sooner or later, and we need never fear but that He will appear with healing on His wings.

In the meantime we must light our own lamps, and keep them trimmed and burning with the oil of God's Holy Spirit, while we watch and wait for the coming of our Lord. Not like the foolish virgins who fell asleep while they waited, and let their lamps go out, so that when the bridegroom appeared, it was too late to buy more oil. How foolish we are! because we need only ask, and our loving Father will give us the oil without money, and without price. We repay Him with love and obedience.

Often unhappily, we cannot see we are blind men walking in darkness, full of grief because the light of heaven is hid from us by our infirmities. Christ, however, has come, especially has He come to cure such. Try to see Him with the eyes of faith. Let the earnest and only prayer of the blind man, whom Jesus commanded to be brought to Him, rise from the midst of your hearts, "Lord, that I may receive my sight." Persevere, and He will give it you as He did to blind Bartimeus. Then you will see the brightness of His glory, and be cheered by the light of His countenance, and having once seen, keep your eyes steadfastly fixed on your Saviour, nor turn them away lest they behold vanity.

### Medical Applications of Electricity.

*(Concluded from page xcii.)*

"Now I want to show you that sensation is not the right thing to trust to in this kind of work. Here we have a patient who has not been electrified before, and who is rather nervous, so he will tell us all about his sensations. On his forehead he feels a sensation with five cells, on the back with the same number of cells he does not feel it all. Sensation is not to be relied upon, because it varies in different parts of the same body, it also varies in the same parts of the body in different individuals, this is true in health, and is still more true in pathological conditions, where the patients may be unable to feel an enormous current, or may suffer acutely under a very small one.

Let us now consider the methods of administration of electricity. In central galvanisation we use the continuous current, applying the negative pole to the epigastric region, and using the positive pole to the forehead, head, neck, and spine in succession. This is a very useful method. I will demonstrate it upon a subject. Let us assume that I am to give a maximum of 1½ milli-ampères to the head, 5 to the neck, and 10 to the spine. We might, without much harm, go up to 20 milli-ampères on the back, but as the patient is nervous we shall not do so. To give 1 milli-ampère to the head, not many cells are wanted, but they may be increased as required for the other parts. We place the positive pole in the form of a moistened sponge on the patient's head, beginning with the forehead, and allow barely the 1 milli-ampère of current to pass. The head is a delicate portion of the system, and must not be too strongly electrified. Passing to the centre of the crown of the head, we moisten the hair to increase its conductivity, increase the number of cells, and pass the current through the brain for another two minutes. We proceed round the moistened head, keeping in the centre, because transverse currents in the brain are unpleasant, and may be dangerous; they should never be given, unless under clear and definite instructions from a medical man. Coming to the nape of the neck, we increase the current to 5 milli-ampères and apply it for another three minutes. Then proceeding to the spine, we take it in sections, and sponge it all the way down. This sponging up and down the spine should last for five minutes, and the number of cells should be increased so as to give a current of 10 milli-ampères. Please note that though I began with 5 cells to produce 1½ milli-ampères, I am now in this part of the body obliged to use 24 cells to produce 9 milli-ampères. This again proves the fallacy of trusting to a given number of cells. Had I used 10 or 15 cells throughout I should have given a serious overdose to the head and an absurdly small dose to the spine." In reference to general Faradisation, or Flow-ruption, as he preferred to call it, the lecturer said the need for measuring the alternating current is not so great as for the continuous current, for the alternating current has little, if any, power of electrolysis and cataphorisis. It is further very difficult to measure the alternating current in sufficiently small doses for medical purposes. It produces so much muscular contraction that even one milli-ampère can seldom be borne. I am anxiously looking for the time when we shall be able to measure the alternating current in micro-ampères. A convenient form of approximate measurement is that suggested by Du Bois Raymond, and used by this institute, in which the relative distances of the secondary and primary coils from each other is taken as an indication of the current passing.

Mr. Lawrence then demonstrated upon another subject various methods of local application to a limb and to the face; and further, by means of a model bath and a large doll, illustrated several ways of administering electric baths. He deprecated the practice of electrifying the water only, on the ground that as the water generally has less resistance than the body nearly all the current supplied passes through the water, from electrode to electrode, and very little, if any, enters the body of the patient. He preferred to apply one electrode to the neck, just outside the water, the other alone being placed in the water, as by this means the whole of the current must pass through the patient's body, and a measurable dose be administered.

In conclusion, the lecturer spoke of the need for attention to details in medical applications of electricity and of the necessity for a careful study of electricity—its various actions, its methods of control, and measurement—before attempting to apply so powerful an agent to so delicate and complicated a structure as the human body.

### Nurses on Their Travels.

### BUENOS AYRES.

Having seen an enquiry from "Muriel" in a number of your valuable paper, forwarded to me out here, respecting nursing in Buenos Ayres—perhaps some information from one who is at present out there, and who can speak from personal experience, may be useful to nurses who contemplate taking the rather serious step of coming out here without fully understanding to what they are coming. Firstly, let them realise that this is not an English colony, but a Spanish Republic, and although there is a very large English-speaking community, the language spoken everywhere is Spanish. There are a very considerable number of English nurses out here, and they have greatly increased in number during the last year or so. I see many advertisements in the daily papers of nurses requiring work. The pay sounds good: 30 dols. or 35 dols. a-week; but against this you have to remember that the currency is paper, which is always fluctuating in value, and at present, owing to various causes, it is much depreciated. The nominal value of the paper dollar is 4s. 2d.; at present its value is about 1s. 6d. The price of living is enormous, and all other prices are proportionately high. My advice to those who think of coming out here expecting to make more than they do at home, is much the same as "Punch's" advice to those about to marry—"Don't"; but if they are determined let them be certificated in every branch of their calling, possess a midwifery diploma, and have a fair knowledge of Spanish, and consequently able to take their Spanish diploma out here, without which they cannot practise.

The British Hospital has no vacancies. The Matron, Miss Eames, and her staff of nurses came out from St. Thomas's, and owing to want of funds and accommodation, the number cannot be increased. The climate is bright and clear, intensely hot from December till the end of February; fairly healthy. New comers generally seem to have to go through a process of acclimitisation in the form of diarrhœa or typhoid Flannel should be worn to guard against chills, as the variations are very great; otherwise in the summer clothing should be as cool as possible. Laundry expenses are very heavy, and the linen is, as a rule, very indifferently got up. Cuffs and collars should be dispensed with, and a washing edging substituted. Personally, I like the country very much; but let no nurse think she can make her fortune out here quicker than she can at home, unless she is prepared to take her Spanish diploma. Any nurse must be prepared to act without medical aid out here; in some cases there may not be a doctor within many leagues. "Veritas."

### Examination Questions.

THE answers received to this month's examination questions were comparatively few, probably owing to the rush of Christmas entertainments. The best answer was undoubtedly that of Sister Mary Gardner, had she not unfortunately gone beyond the point in question, and discoursed on cold packs and sponging. As our question dealt solely with the cold bath we have awarded the prize to Nurse Emma Andrew, of the London Hospital. Amongst the answers deserving honourable mention are those from Nurse Ada Whiteman, Nurse Maggie Stocks, Nurse Emily de Levanté, Nurse A. Gough, and Nurse Ethel Wilson. The question for February is, "How would you apply, and dress, a blister?" Answers must reach this office by February 10th; they must be short and concise, and written on one side of the paper only.

PRIZE ANSWER FOR JANUARY.—How to give a cold bath if ordered for hyperpyrexia:—"The patient's clothes are removed, and either a gown which is kept for the purpose or a sheet is put around him. In case of typhoid it is a good plan to put a binder around the abdomen, in order to give support. The bath should be drawn close to the bed and the patient gently lifted in by, at least, four people. The temperature should be about 98 deg. Fahr. One arm is left exposed, so that the medical man may be enabled to feel the pulse the whole time. Cold water or ice (sometimes salt) is then added till the desired temperature is obtained, the bath thermometer being constantly used. The patient's temperature must be taken frequently. The time for remaining in the bath varies; sometimes the patient cannot stand it more than eight or ten minutes, and sometimes may for two hours. A nurse should have at hand besides the bath a clinical thermometer, brandy or milk, measure glass, and spoon, in case of the patient getting collapsed. The brandy syringe might be useful. Whilst the patient is in the bath, macintoshes must be placed on the bed, blankets made hot, and hot-water bottles got ready for the feet. When he is taken out of the bath he should be placed in blankets for a little time, then have the wet things removed and the warm bedding replaced."

### Presentations.

THE nurses of the Cambridge Nursing Home have presented their Lady Superintendent, Miss C. M. Löhr, with a handsome gilt carriage clock in morocco case, and with a suitable inscription engraven upon it, on the occasion of her resignation. The gift was much appreciated by Miss Löhr, who was deeply gratified by the kind feeling of her nurses. Her departure is a great regret to all, for her amiable and gentle disposition had won her universal esteem and love. Her sister, Miss A. L. Löhr, was also presented with a handsomely bound volume of Longfellow's poems, as a token of gratitude from the nurses, to whom she had ever been most kind.

THE PRESTON AND COUNTY OF LANCASTER ROYAL INFIRMARY.—On the evenings of the 7th and 20th of January, two interesting presentations were made by the matron superintendent of this infirmary in the female ward, to Nurse Leslie, for six and a-half years head nurse of that ward (who is now resigning her present post for that of matron of the Nurses' Home in connexion with this institution), on behalf of her patients and nurses. The first presentation, which was held on the 7th, consisted of a pair of Worcester vases, given by the patients as a token of respect and affection; and the second presentation, which was held on the 20th, consisted of a handsome piece of plate, which took the form of a Queen Anne tea-kettle and stand, given by the nurses, in token of their appreciation of her valuable training, and of the high esteem in which she was held by them.

### Everybody's Opinion.

[*Correspondence on all subjects is invited, but we cannot in any way be responsible for the opinions expressed by our correspondents. No communications can be entertained if the name and address of the correspondent is not given, or unless one side of the paper only be written on.*]

### PRINCESS CHRISTIAN'S DAUGHTER.

"A MATRON" writes: I am much pleased that it is proposed to give the Princess Christian's daughter a present from nurses on her marriage. Some plan should be settled upon at once and made public, so that matrons, sisters, and nurses may know how to proceed. I should suggest that some well known person in the nursing world should be asked to receive donations, and that the sums shall be fixed, say matrons 5s., sisters 2s. 6d., nurses 1s., which shall not be allowed to be exceeded, although less in each class will be accepted. Would it not be well to open a subscription list immediately, and close it on a certain date, and when the amount is known the Princess Christian should be waited upon to decide what form the presentation shall take.

[We are happy to endorse this suggestion. Miss E. Durham, Farringford, Freshwater, has consented to act as Secretary, and all subscriptions sent to her will be duly acknowledged in THE HOSPITAL. The subscription list will be kept open for one month from this date, and those who desire to join in the movement should send their subscriptions to Miss Durham at once.—ED. *T. H.*]

### NURSES AND INFECTION.

MR. W. H. PEARCE (Secretary of the Paddington Green Children's Hospital) writes: With reference to the letter which appeared in your paper of the 24th instant under the above heading, I am directed to state that the complaint suggested by Mrs. Phillips' letter against this hospital, is receiving the most careful investigation on the part of the Committee and staff; but that so far as the letter referred to reflects on the Matron of the hospital, the Committee desire to express their emphatic dissent from it.

### Notes and Queries.

### Queries.

(29) *Air Cushions.*—Where can round silk air cushions be bought?—*D. A. T.*

(30) *Red Cross Pincushion.*—Where can I buy a ready-made Red Cross pincushion? I have applied to the Sussex County Hospital, but they no longer sell them.—*Nurse N.*

(31) *Knitted Petticoats.*—Will anyone give a disabled nurse orders for knitted petticoats?—Miss Wilson, 45, Colville Gardens, Bayswater, W.

(32) *Board and Lodging for a Private Nurse.*—*Nurse F. writes:* Can any reader of THE HOSPITAL inform me where a private nurse (not a lady), can have board and lodging at a reasonable rate? Must be in London.

### Answers.

(25) *Washing Blankets.*—Boil half a pound of common yellow soap in two gallons of water, add two tablespoonfuls of paraffin oil; boil the whole quickly for five minutes. Let stand till warm, put blankets in without rubbing them, and the dirt will all rise to the top. Rinse in four clean waters to get rid of smell. For further particulars write to E. Phillips, 90, Bolton Road, Manchester.

(27) *Attendants.*—Advertisements for attendants are generally inserted in the first column of the *Daily Telegraph* or in the county newspapers.

(28) *Home Wanted.*—Apply to the Cottage Hospital, St. Paul's Cray. See answers to 26.

*Subscriber.*—Write to the Hon. Secretaries, the Hospitals Association, 140, Strand, W.C. It is ridiculous that hospital people should be so chary of asking help and advice of one another.

*Thistle.*—We think the subject had better drop for the present; we understand steps will shortly be taken to remedy matters.

*L. N.*—The Junius S. Morgan Fund is only available for those nurses who belong to the Pension Fund, or for nurses over sixty years of age. The whole effort of the Fund is to help those who help themselves, and you had better join it at once; no matter for how small a sum.

*Newly Elected.*—You will find in "The Hospital Annual" a complete method of keeping all your books—both housekeeping and others. We congratulate you, and hope you will find it easier than you expect to combine the duties of Matron and Secretary. You will find the book-keeping very easy, and easily understood if you follow the "Annual" method.

*Nurses' Co-operation.*—The charge for nurses living at 8, New Cavendish Street, will be 16s. a week, or 3s. a day.

### The World Below the Diaphragm.

"Now, Doctor, come and please explain these coils and complications,
I've suffered long (below the ribs) and merit explanations;
For, what with bile, and block, and pain, full stop—or semicolon,
It's quite enough to turn the brain of Socrates or Solon.

So look beneath the diaphragm, unravel me these wonders,
For I'm half mad with torture sad and making many blunders.
We'll leave the thorax quite alone, for regions more outspoken,
My lungs are sound, and, strange to say, my heart is still unbroken."

So, starting with the stomach straight, ignoring spine and marrow,
We stuck at the pylorus first, on finding it so narrow;
Then explored the small intestine, with its manifold gyrations,
How to liver, gall-bladder, and duct, it bore its own relations.

From the duodenum onwards, through its curves and genuflexions,
Tracing over its circumference and front and back connections,
And in winding round the ileum we nearly lost our way,
And at the ileo-cæcal valve we met with sore delay.

Then up the wider colon—sure the race was nearly run—
And the sigmoid-flexure safely passed I thought the perils done,
For the rectum foretold straightness—but alas for such a craze,
I found it curved like all the rest of this bewild'ring maze.

"But now—the 'appendix-vermiform'—pray say what it may mean,
It puzzles more than pancreas, or liver-lobes, or spleen;
The use of it I cannot grasp (though long I've tried in vain),
Except to catch a cherry-stone, and make a nasty pain!"

"Oh, that's a relic of the time ere human life began,
It shows that we have risen by steps through mammals up to man.
It's like the coccyx-bone, you see, the curve that ends the spine
(A legacy of tail is this, straight from the monkey line).

It helps the Darwin theory—a ladder-like ascent,
From low to highest ever is the path that nature meant;
Development's the law of life—our studies prove it so—"
To this I humbly bowed my head and meekly uttered "O!"

But "survival of the fittest," why, 'tis surely most unfit
To leave a process here to catch the cherry-stones in it!
I will ponder o'er these problems, and will come another day;
We're not far up the ladder yet is all that I can say.

So, what with veins and arteries, and pancreas and spleen
(This last, perhaps, a legacy from Cain, or Ahab's queen),
What with liver-lobes, and kidneys, there's such a heap to see,
That the World below the Diaphragm is far too much for me!

B. G.

### What to Read.

One of the latest volumes of the Camelot series is "Shorter Stories" by Balzac, excellently translated by William Wilson and Count Stenbock. These stories are very eerie, and the very model of what short stories ought to be; a shilling would be well expended by a nurse on this volume. "Women Poets of the Victorian Era" is another shilling book worth buying, though it is not well edited; there are all sorts of mediocre verses by unknown writers, and only a poor selection from the poems of Mrs. Barrett Browning, George Eliot, and Graham R. Tomson. Still, the volume is handy and cheap, and contains many a page calculated to cheer and help the weary nurse. The *Argosy* for January contains an interesting article on Kate Marsden. The last volume of the Schopenhauer series, "Studies in Pessimism," contains the celebrated diatribe against women. It is sometimes well to see life with the eyes of the weary and disgusted philosopher, so perhaps some of our readers may care to spend their half-crowns on this book. Cassell's National Library is coming out in a re-issue, vol. I., "The Haunted Man," by Dickens, price 3d. Professor Drummond has issued another little white and gold pamphlet this year, "Pax Vobiscum," it is needless to say it is well worth buying and studying, price 1s. Books to be had from the library are "Dreams," by Olive Schreiner, a beautiful volume of allegories; "Kestell of Greystone,' an interesting and pathetic three vol. novel, by Esmé Stuart; and "Sister Philomène," by the Brothers De Goncourt, translated by Miss Laura Ensor. This last is the story of a religious nursing sister and an atheistic doctor. It is well written, and the plot powerfully worked out; the illustrations also are excellent.

### Impromptu.

"We have always looked upon professional nurses as a strange compound between a nun and a barmaid."—*Vide Comic (?) Paper.*

A Nurse! "Strange compound," so a cynic tells
"Of nun and barmaid," and on this he dwells;
Thinking thereby, with meaning smile and sneer,
To mark his wit and strike a blow severe.

A nun! fit emblem of devoted zeal,
Toil without ceasing for another's weal,
Of holy labour, and of self no thought,
Severe the struggle, high the guerdon sought.

A barmaid! truly, if *we* judge aright,
A nurse (as barmaid) should be cheerful, bright;
Doing her duty (more we ask of none),
Dispensing comforts —— Has the cynic done?

A nurse! who soothes the fevered brow with care,
Relieves our pain, nor dreads infection's snare:
Can man be found who would to such deny
Their mead of praise, and on a sneer rely?

Smile gaily, cynics, while in health you may,
When sickness threatens there will come a day
When *you'll* be glad to praise in very truth
A nurse, "'twixt barmaid and a nun" forsooth!

Adam.

### Amusements and Relaxation.

### SPECIAL NOTICE TO CORRESPONDENTS.

First quarterly word competition commenced January 3rd, 1891; ends March 28th, 1891.

Competitors can enter for *all* quarterly competitions, but no competitor can take more than *one first prize* or *two* prizes of any kind during the year.

Three prizes of 15s., 10s., 5s., will be given for the largest number of words derived from the words set for dissection.

Proper names, abbreviations, foreign words, words of less than four letters, and repetitions are barred; plurals, and past and present participles of verbs, are allowed. Nuttall's *Standard* dictionary only to be used.

N.B.—Word dissections must be sent in WEEKLY not later than the first post on Thursday to the *Prize Editor*, 140, Strand, W.C., arranged alphabetically, with correct total affixed.

The word for dissection for this, the FIFTH week of the quarter, being "ACONITE."

| Names. | Jan. 22nd. | Totals. | Names. | Jan. 22nd. | Totals. |
|---|---|---|---|---|---|
| Reynard | — | 77 | Crystal | 51 | 121 |
| Reldas | 70 | 146 | Woodbine | — | 25 |
| Tinie | — | 30 | Madame B. | — | 25 |
| Patience | — | 76 | Shakespeare | — | 59 |
| Jenny Wren | 63 | 135 | Smyrna | 40 | 83 |
| Agamemnon | 62 | 138 | Southwood | 34 | 77 |
| Wyamaris | 65 | 138 | Gipsy Queen | — | 21 |
| E. C. | 67 | 142 | Snowball | — | 19 |
| Ecila | 66 | 138 | Rita | 52 | 88 |
| Hope | 67 | 142 | Mortal | — | 16 |
| M. W. | 67 | 142 | Nurse Annie | — | 15 |
| Qu'appelle | 67 | 142 | Carmen | — | 11 |
| Nil Desperandum | 62 | 137 | Grannie | 45 | 45 |
| Lady Betty | 56 | 130 | Amie | 30 | 30 |
| H. A. S. | 52 | 120 | M. R. | 25 | 25 |
| Sister Jack | 36 | 62 | Primrose | 24 | 24 |

### Notice to Correspondents.

N.B.—Each paper must be signed by the author with his or her real name and address. A *nom de plume* may be added if the writer does not desire to be referred to by us by his real name. In the case of all prize-winners, however, the real name and address will be published.

## Figures and Tables

**Figure f1:**
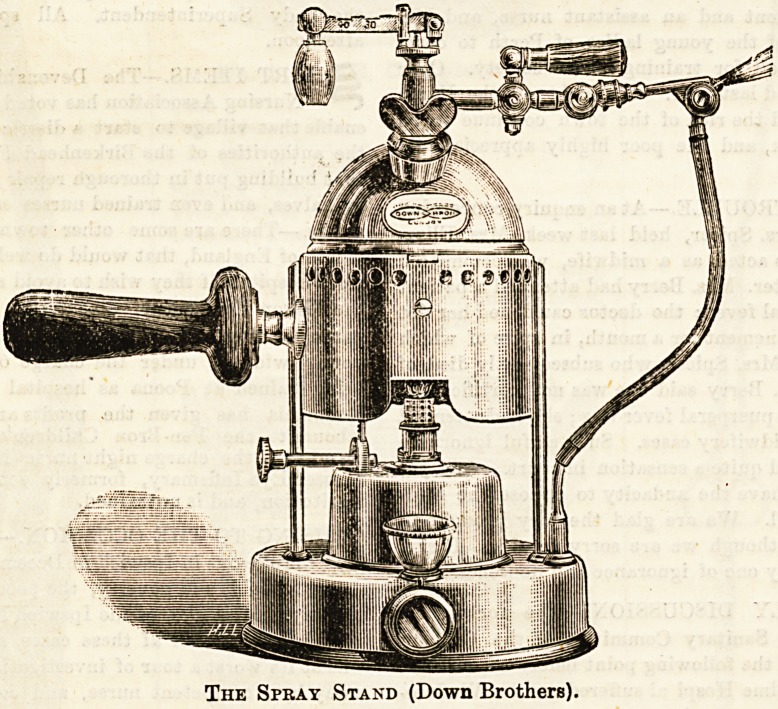


**Figure f2:**
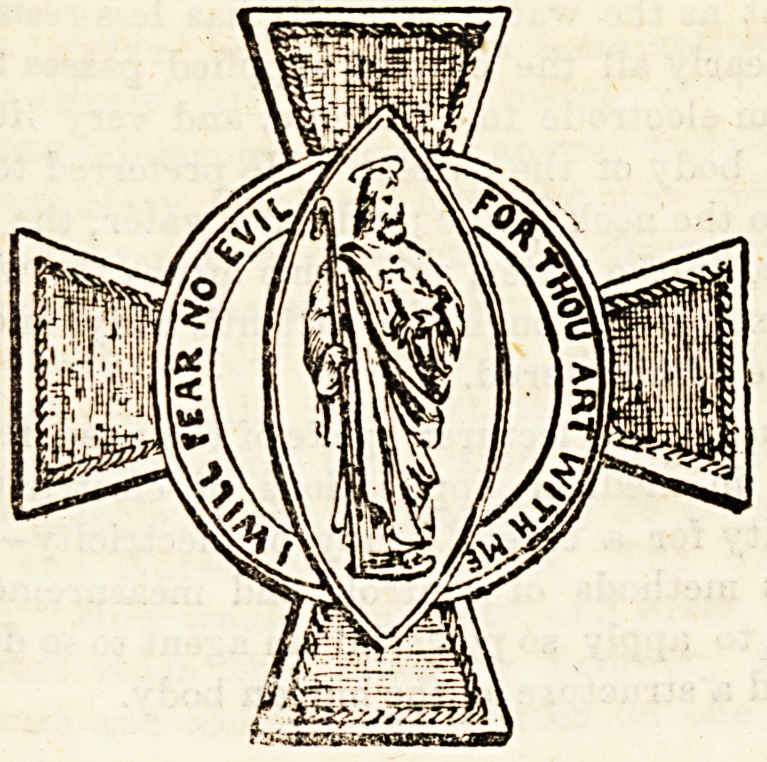


**Figure f3:**